# Bottom-up formation of robust gold carbide

**DOI:** 10.1038/srep08891

**Published:** 2015-03-16

**Authors:** Benedikt Westenfelder, Johannes Biskupek, Jannik C. Meyer, Simon Kurasch, Xiaohang Lin, Ferdinand Scholz, Axel Gross, Ute Kaiser

**Affiliations:** 1 Institute of Optoelectronics, Ulm University, 89081 Ulm, Germany; 2 Central Facility of Electron Microscopy, Ulm University, 89081 Ulm, Germany; 3 Department of Physics, University of Vienna, 1090 Vienna, Austria; 4 Institute of Theoretical Chemistry, Ulm University, 89081 Ulm, Germany

## Abstract

A new phenomenon of structural reorganization is discovered and characterized for a gold-carbon system by *in-situ* atomic-resolution imaging at temperatures up to 1300 K. Here, a graphene sheet serves in three ways, as a quasi transparent substrate for aberration-corrected high-resolution transmission electron microscopy, as an in-situ heater, and as carbon supplier. The sheet has been decorated with gold nanoislands beforehand. During electron irradiation at 80 kV and at elevated temperatures, the accumulation of gold atoms has been observed on defective graphene sites or edges as well as at the facets of gold nanocrystals. Both resulted in clustering, forming unusual crystalline structures. Their lattice parameters and surface termination differ significantly from standard gold nanocrystals. The experimental data, supported by electron energy loss spectroscopy and density-functional theory calculations, suggests that isolated gold and carbon atoms form – under conditions of heat and electron irradiation – a novel type of compound crystal, Au-C in zincblende structure. The novel material is metastable, but surprisingly robust, even under annealing condition.

In 1900, Mathews & Watters reported about a very explosive gold acetylide Au_2_C_2_^1^. The substance has been treated as a ‘true’ carbide and it was indeed the first time that the existence of gold carbide has been reported ever. Today, more than hundred years later, the study of various compounds containing gold carbon bonds is covered by the field of organogold chemistry^2^. However, in comparison to many other pure metal carbides, there is no experimental evidence for a possible inorganic crystalline gold carbon compound and its structural properties^3^. This is not surprising, because bulk gold has almost no solubility for carbon under equilibrium conditions and the only (above mentioned) gold carbide supposed to be crystalline turned out to be extremely unstable^4^. Nowadays, there are several successful approaches in synthesizing carbide clusters from late transition metals including gold^3^. However, theses clusters are electrically charged and consist of only a very small number of atoms. Furthermore, several concepts have been developed in synthesizing, characterizing and understanding metastable carbide crystals regarding transition metals like cobalt, palladium and nickel^5^,^6^ but still with the exception of gold.

The structural analysis of new materials has undergone a tremendous improvement in the capabilities to explore the atomic configuration by aberration-corrected high-resolution transmission electron microscopy (AC-HRTEM), in particular due to the successful correction of lens aberrations^7^,^8^. It is now possible to obtain atomic-resolution images even for light-element materials such as carbon, nitrogen, and oxygen, with a minimization of atom knock-on damage by using reduced acceleration voltages^9^,^10^. The understanding of carbon systems such as graphene or carbon nanotubes has significantly benefitted from this development. *In-situ* experiments enable the study of dynamic phenomena under direct observation of the atomic structure^11^ within the range from room temperature (RT) up to ca. 2000 K^12^. Therein, the potential of a free-standing graphene membrane as transparent and heatable substrate has been already demonstrated. Furthermore, this approach could be applied to investigate the transformation of carbon adsorbates into graphene with atomic resolution^13^.

In the present work, the approach of in-situ heating via electrical biasing the graphene has been applied with focus on gold nanoislands and their interaction with the graphene substrate driven by irradiation. Surprisingly, the bottom-up formation of a novel, very robust gold-carbon compound has been observed, as further evidenced by electron energy loss spectroscopy (EELS) and the help of density-functional theory (DFT) calculations.

## Results and Discussion

### Sample preparation

For this work, free-standing graphene layers with electric contacts in a TEM-compatible geometry have been prepared. This concept has been optimized for combining Joule heating experimentation via applying an electrical current to a graphene sheet with real-time atomic-level imaging. The configuration of the electrically contacted sample cartridge and temperature estimates have been described in detail previously^12^.

In brief, a mechanically exfoliated graphene sheet is transferred onto a Si/SiN membrane structure with open windows and gold contacts, such that an electrically contacted and completely free-standing few-layer graphene substrate in a TEM-compatible geometry is formed. Gold nanoislands have been deposited ex-situ via thermal evaporation onto graphene. Their melting temperature provided an estimate of the local temperature. Here, additionally in-situ TEM has been performed applying AC-HRTEM combined with local electron energy loss spectroscopy (EELS) (further details are described in the methodological section below).

### In-situ HRTEM and HRTEM image analysis

Even though, the original interest of this experiment was only the thermally driven interplay between the gold nano particles and the graphene substrate, it turned out, that the electron irradiation necessary for TEM imaging influences the interaction between gold and carbon in a very unexpeted manner. Depending on the local heating temperature, this aspect is very important for the reactions that will be described below.

The diffusion of gold adatoms on the nanoislands is already significant at RT^14^,^15^ and led to continuous shape changes of the particles upon heating^16^. After exceeding a certain temperature limit, the first almost spherically shaped particles form liquid drops and start to evaporate^12^,^13^,^17^. In accordance with theoretical predictions and similar experiments, the related melting temperature strongly depends on the particle size^18^ and in this work, is estimated to be in the range of 800 K to 1300 K for particle diameters in the range of 3 to 20 nm. The observations described in the following occurred at temperatures closely below the melting point of the gold particles (i.e. just before the particles melted upon increasing the heating current).

**Figure 1:**
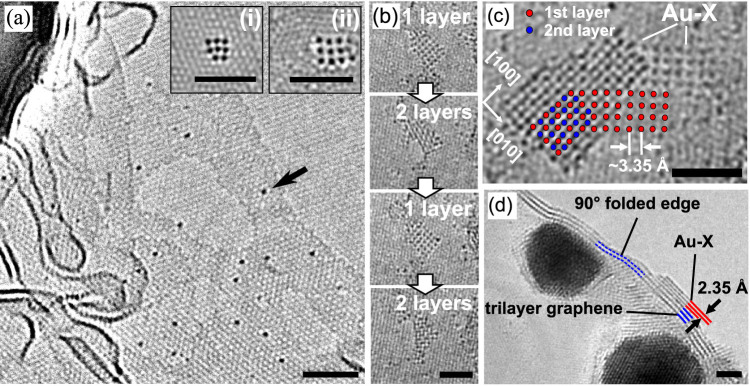
AC-HRTEM images at 80 keV showing the atomic arrangements of gold atoms explored from tiny clusters towards small crystallites (scale bars are 2 nm). (a) Isolated heavy gold atoms trapped in carbon contamination on graphene, example indicated by an arrow. A larger gold particle is present in the upper left corner. Insets (i, ii) show small clusters that form under irradiation and heat within the carbon contamination layer on top of graphene. (i) One very rare example found to be consistent with the interatomic distance of traditional gold clusters. Here, it has been determined to be 2.6 Å. (ii) One of the frequently observed clusters having a larger spacing of 3.35 Å. (b) A repeated and abrupt switching of a mono-layer and bi-layer structure in the same position. (c) A connected single- and double-layer region. The circles illustrate the atoms of the first and the second layer, respectively. (d) A few-layer graphene sheet with edges folded into a 90° angle. This side view enables occasionally also the side view on the Au-X structure (see marked region where 3 folded Au-X layers are found on top of folded 3-layer graphene.

The diffusion barrier for gold adatoms on graphene is known to be fairly low- theoretically estimated to be 0.05 eV^19^ – allowing the gold atoms to diffuse very easily along the surface^19^,^20^. Considering the average time used for a single frame exposure (0.5 to 1.0 s), it is impossible to observe the movement of the adatoms directly by TEM. For this reason, single gold atoms could be only visualized, when they got trapped at grain boundaries, edge reconstructions or vacancies^21^. This confirms recently published DFT calculations concerning in-plane adsorption on vacancies and the surface diffusion of gold atoms adsorbed on graphene^20^,^22^,^23^. However, in those studies there is no consideration of electron irradiation-induced diffusion mechanisms as discussed by Urban & Seeger^24^ and Banhard^25^. During a thermally driven crystallization process of adsorbed amorphous carbon layers^13^, plenty of gold atoms have been found, which were trapped and incorporated into such lattice irregularities or grain boundaries (see Figure 1a).

In many cases the gold atoms incorporated into the adsorbed amorphous carbon layers form little clusters with quadratic arrangements (insets of Figure 1a). Typically, the clusters exhibit a surprisingly large nearest-neighbor atomic distance of 3.35 Å (see Figure 1a (ii)). Considering their ultra small size and therefore induced deviation of the lattice parameter, only one example could be identified that coincides with the bulk value of gold (2.88 Å, see Figure 1a (i))^26^. It has been assumed initially that the larger spacing is due to the incorporation of other low atomic number species into the 2-D gold lattice. Unfortunately, the prevailing imaging conditions do not allow the visualization of light elements like carbon in-between the heavy gold atoms (see discussion based on image simulations below).

Several regular monolayers of Au-X with the 3.35 Å spacing have been observed, but only up to a size of about 30 Au-atoms, whereas larger Au-X clusters form multi-layer structures (Figure 1b, c). In some cases, the transitions between single and double layers could be followed directly in real time (Figure 1b). Figure 1c shows a different example, where only a part of the structure is a single layer and the other part is a double or triple layer.

**Figure 2:**
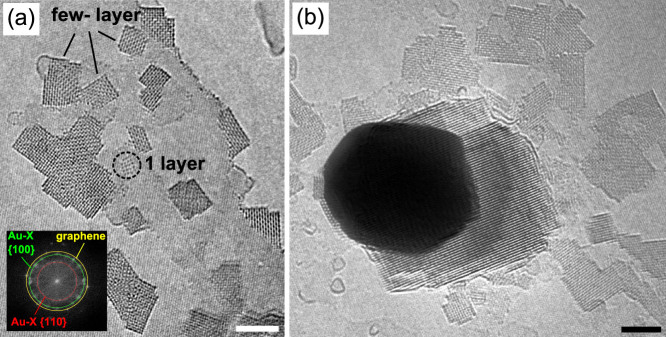
AC-HRTEM images at 80 keV of larger compound crystals formed under ongoing heat treatment and irradiation. (a) Overview image showing 1–2 nm sized cubes of the Au-X structure, and one example of an Au-X mono-layer arrangement. The Fourier transform (inset) provides an accurate estimate of the lattice spacings, using graphene as calibration standard. (b) A thicker example of the new structure growing directly at a gold particle, which may serve as a seed and gold feedstock. A non-linear contrast scale was applied here to show thick and thin structures in the same image. All scale bars are 2 nm.

A side-view of the structure could be obtained for a cluster that had formed on the curved edge of a (partly broken) few-layer graphene substrate (Figure 1d). Broken edges of freestanding graphene and few-layer graphene tend to curl up and show the typical van der Waals distance of 3.35 Å between the layers^27^,^28^. A distance of 2.35 Å could be measured between the first and the second layer of the Au-X structure as marked in Figure 1d. Assuming that the atoms in the second layer are placed above the gaps of the first layer atoms (as shown in the plan-view in Figure 1c), the same Au-Au spacing of 3.35 Å has been obtained as between atoms inside a layer. Figure 2 shows a tiny single-layer example along with several larger, multi-layer structures. Remarkably, the 3-D objects form precise cuboid shapes, as evidenced by their constant thickness (same contrast throughout the rectangle-shaped projection).

Mostly, the cuboids can be found closely to the normal gold nanoparticles whereas the largest ones have been found even directly attached to one of those (Figure 2b), showing a preference for the (111)-facets (see also sequences M1a, M1b and the snapshot in Figure 1 of the supporting information). Growing Au-X structures do not just collect additional gold atoms, but seemingly ‘eat’ partly crystallized layers of carbon or tiny fullerenes in order to increase their capacity of gold atoms at the same time (M1b and M2 in the supporting information). Furthermore, the growth and disappearance of those tiny objects at the boundaries of holes in the few-layer graphene substrate has been observed atom by atom (sequence M2 and at end of sequence M3 in the supporting information). Interestingly, this novel kind of material seems to be relatively stable under the electron beam, but nevertheless it further reorganizes under its influence (M2). Single larger objects tend to decompose and form several new smaller particles after increasing the electron dose rate typically from 5·10^5^ *e^−^*/nm^2^s^1^ to 3·10^6^ *e^−^*/nm^2^s^1^. Another interesting observation was the continuous crystallization process into a small cube beginning from a fully amorphous droplet (M3 and in M1 at the upper left facet).

From the top- and side-view images, and the single- to multi-layer transitions, the structure of the Au part of the lattice can be established unambiguously. The gold atoms of these 3D nanocrystals exhibit a face-centered cubic (fcc) lattice structure with a corresponding lattice parameter of √2·(3.35 ± 0.1) Å (a precise estimate was obtained from the Fourier transformation (FFT) in Figure 2a taking the underlying graphene lattice as a calibration reference). Bulk gold also has a fcc structure but with a smaller lattice parameter of √2·2.88 Å.

**Figure 3:**
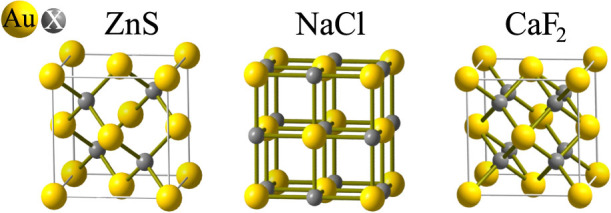
Possible crystal structures with an unknown element X in the octahedral or in the tetrahedral voids of the gold fcc lattice.

As already mentioned, the large nearest-neighbor atomic distance suggests the formation of a chemical compound, where another element is embedded into the voids between the Au atoms. To clarify the amount of visibility of a potentially present second element, corresponding HRTEM image simulations has been performed on flat layers exhibiting a fcc lattice based crystal structure, such as illustrated by Figure 3. Indeed, the result shows that for light elements such as carbon or even for silicon, it is hard to spot the second contributing element under the prevailing imaging conditions (see Figure S9 of the supporting information). As second element, elements have been considered which are else present in the sample (silicon, nitrogen, carbon), or reasonably present in the contamination (carbon, oxygen, silicon and potassium, the latter was used in the sample transfer process)^29^. First of all, the EELS analysis has been described below in order to clarify the presence or absence of these elements. Then the results of DFT calculations have been described in order to find matching lattice spacing and crystal structures. Three different crystal structures are compatible to the observed fcc lattice for the incorporation of a second element: These are the zincblende (ZnS), the rock-salt (NaCl) and the fluorite structure (CaF_2_).

### Electron energy loss spectroscopy

**Figure 4:**
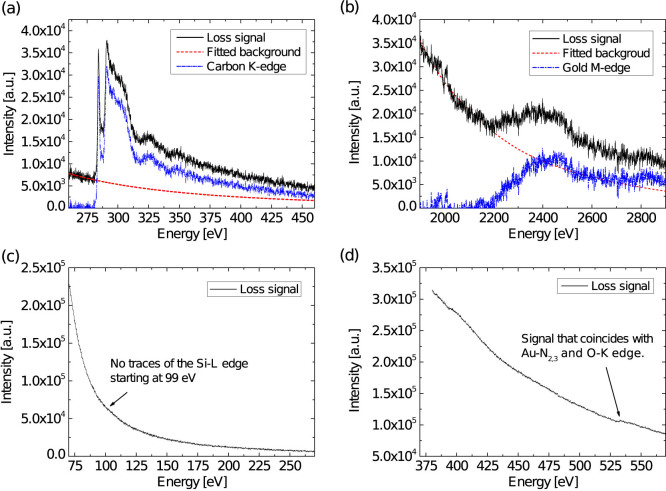
Local EELS spectra acquired from Au-X on top of graphene. (a) Original energy loss and extracted carbon K edge at 284 eV (b) Original energy loss and extracted gold M_5_ edge at 2206 eV. (c) EELS spectrum obtained within energy range from 80 to 280 eV showing no signal of the Si-K edge at 99 eV. (d) EELS spectrum obtained within energy range from 370 to 570 eV, the signal at around 540 eV could originate from gold (N_2,3_ edge) or oxygen (K edge).

In order to determine the second element, local EELS was carried out in the TEM using a small beam diameter (5 nm). Multiple spectra with different energy ranges were acquired to consider the energy windows of the absorption edges of the above mentioned elements. Figure 4 shows the EELS measurements acquired from a single Au-X cuboid on top of graphene (see Figure S8 in the supporting information). The presence of the elements carbon (Figure 4a) and gold (Figure 4b) is indicated by the clear signals of the carbon K absorption edge at 284 eV and the gold M_5_ edge at 2206 eV. The EELS data show that silicon (Figure 4c), nitrogen, and oxygen (Figure 4d) as constituents can be excluded as the local EELS signals do not show any corresponding absorption edges. The small signal at around 540 eV originates most probably from gold (N_2,3_ edge). Energetically, it would also fit to the oxygen K edge. However, this signal is too weak (intensity of signal only 0.1% above background) and therefore it would only correspond to a handful of atoms^30^. Although the detection of carbon appears to be trivial, because the Au-X cuboid is directly located on top of the multilayer graphene substrate, the exclusion of all other reasonable elements justifies the conclusion that carbon must be the only constituent element in addition to gold.

Furthermore, it has been found, that these ‘Au-X’ cuboids survived the exposure to ambient conditions. This has been tested by subsequent annealing of the entire sample on a hotplate at 200°C in ambient air. Their thermal stability in the vacuum of the TEM column appears to be at least as high as that of the gold nanoparticles or even higher: Although the evaporation of the neighboring gold nanoparticles has been already started, the cuboid structures start to decompose only upon further heating. When this happens, it occurs directly, i.e. no liquid phase in-between has been observed (sequence M4 in supporting information). The ‘Au-X’ cuboids have first been found accidently, i.e. without any purpose, in 3 independent experiments of heating gold-decorated graphene. In two further experiments the in-situ growth of the ‘Au-X’ material intentionally via Joule heating up to 1000 K has been reproduced, followed by continuous electron irradiation.

### Density functional theory calculations

In order to confirm the EELS interpretation DFT calculations have been performed. Table 1 shows the predicted lattice parameters for each of the three structures (zincblende, rocksalt and fluorite) and four candidate second elements. Clearly, only the small elements from the second row of the periodic table (C, N, O) provide a reasonable lattice match, while e.g. any theoretically considered silicon compound would result in a much larger lattice parameter than observed in the experiment. This also fits to the above mentioned EELS spectra showing silicon not to be locally present. Indeed, the smallest lattice parameter deviation *Δ* is achieved for the NaCl structures of AuC, AuN and AuO. However, DFT calculations combined with the generalized gradient approximation (GGA) tend to yield larger values compared to those observed in the experiments^31^,^32^, i.e. the calculated DFT-GGA value for gold is about 2.4% larger than the experimentally estimated one. If this difference has been treated as a systematic error, best agreement is obtained for the ZnS structure of AuC.

For all structures, also the cohesive energies have been determined, i.e. the compound formation energies with respect to the most stable elemental structure. All calculated cohesive energies are positive. This means that the considered compounds are not thermodynamically stable. For gold carbide, the corresponding energies with respect to bulk gold and graphite in Table 1 indicate that it is most stable in the ZnS structure. The endothermic character of its formation explains why there are so few reports on gold carbide. As already mentioned, it was identified a long time ago, and turned out to be explosive under rapid heating and ignites at temperatures above 180°C^1^. Its structure has not been determined yet. Note, however, that here it is assumed that AuC is not formed from graphite but rather from the hydrocarbon deposits or defects on graphene. Under electron irradiation, both could be considered as permanent source of atomic carbon.

As a further experimental fact, flat crystals and cuboids have been observed with a size not larger than 20 nm and an explicit (100) surface termination. For this reason also the surface energies of thin AuC films with ZnS and NaCl structure have been compared. That calculation is based on a 4 × 4 supercell. For all calculated surface orientations, no significant surface relaxation has been found. In case of the ZnS structure, it has been obtained in agreement with the experiment that the formation of a (100) surface termination should be clearly favored versus the (110) and (111) surfaces (see Figure 1 in the supporting information). Furthermore, the calculation shows that surfaces terminated with gold should be energetically preferred. In contrast to the agreement for ZnS, the (100) surface in the NaCl structure has been obtained to be the energetically most unfavorable one. The calculated electronic structure of a small AuC cluster adsorbed on graphene is only slightly modified compared to the one of the isolated AuC cluster, as shown in the supporting information. This indicates that AuC is weakly interacting with the graphene substrate and retains its properties upon adsorption on graphene. It has been found that AuC is metallic, similar as the stable compound tungsten carbide, WC, whose electronic properties has been determined as a reference. For the bulk modulus of AuC, which has been derived from the DFT-data, a value of 141 GPa has been obtained which is smaller than the bulk modulus of bulk gold, 180 GP^33^. In contrast, the calculated bulk modulus of WC of 338 GPa is slightly larger than the one of tungsten bulk metal, 310 GPa.

From the calculation, there is a reasonable agreement of the lattice structure for the AuC, AuN and AuO compound in NaCl structure, and for an AuC compound in ZnS structure. The surface termination agrees with the experiment only for AuC in a ZnS structure. Hence, the conclusion for AuC is supported by the EELS analysis as well as the DFT calculation. Indeed, there are several additional points that indicate the presence of an AuC compound, rather than AuN or AuO: First, the intermixing of gold into the carbon matrix has been observed on the single-atom level in the heating experiment (Figure 1), so that it is reasonable to assume the same elements also in the few-atom gold clusters. Second, gold oxide and gold nitride have been reported previously in the literature^34^,^35^,^36^,^37^,^38^ to exhibit different stoichiometry and crystal structures. Third, it has been found that the cuboids decompose at temperatures higher than 1000 K. This is in strong contrast to the decomposition temperature known for gold oxide and gold nitride^37^,^38^.

## Conclusion

In summary, atomically resolved *in-situ* TEM studies of the heat- and irradiation-induced formation of novel gold structures on graphene have been presented. Periodic arrangements of individual gold atoms have been found in form of rectangularly shaped atomic monolayers and bilayers as well as entire cuboids of many atomic layers. Both, DFT calculations and EELS analysis provide clear evidence for the formation of a crystalline compound of gold and carbon in a zincblende structure. These AuC nanocrystals are stable under electron irradiation, under ambient conditions, and up to temperatures of ca. 1000 K in vacuum. On the theoretical side, they are predicted to be metallic and exhibit a bulk modulus of 141 GPa. Other properties of this exciting new material remain to be explored.

## Methods

### In-situ TEM

The experiments have been performed applying aberration corrected AC-HRTEM on a TITAN 80–300 FEI microscope at an accelerating voltage of 80 kV. It has been operated with a small positive value of the spherical aberration *C_S_* = 10 μm and underfocus conditions that resulted in black atom contrast. The field assisted Schottky electron emitter was operated with reduced extraction voltage of 2000 V to increase the information limit^39^. The TEM was equipped with a Gatan Quantum GIF 965 energy filter for local electron energy loss spectroscopy. The sample was loaded via a cartridge into a Fischione 2510 biasing holder. An electrical heating current was controlled via a Keithley multimeter and passed through the graphene sample by applying a bias voltage between the electrodes, with a current-density in the range of 2·10^7^ Acm^−2^ at an applied bias of 2 V.

### DFT calculations

The DFT calculations have been performed within the generalized gradient approximation (GGA) for the exchange-correlation functional^40^ using the Vienna ab-initio simulation package (VASP)^41^,^42^. In order to account for electron-ion interactions, the projector augmented wave method (PAW)^43^,^44^ has been used. The electronic one-particle wave functions were expanded in a plane wave basis set up to an energy cut-off of 400 eV. The bulk modulus has been derived by fitting the calculated dependence of the bulk energy as a function of volume to the Birch-Murnaghan isothermal equation of state^45^.

## Supplementary Material

Supplementary Information

Supplementary Information

Supplementary Information

Supplementary Information

Supplementary Information

Supplementary Information

## Figures and Tables

**Table 1:** Calculated lattice parameter *a* and the deviation *Δ* of the lattice parameter with respect to the observed value for various gold compounds and crystal structures. For the gold carbide structures, in addition the cohesive energy with respect to bulk gold and graphite is given

Structure	Compound	a (Å)	*Δ (%)*	*E_coh_* (eV/atom)
ZnS	AuC	4.93	4.1	1.88
	AuN	4.98	5.1	2.17
	AuO	5.11	7.9	2.71
	AuSi	5.58	17.8	-
NaCl	AuC	4.67	−1.4	2.09
	AuN	5.67	−1.4	2.13
	AuO	4.76	0.5	2.58
	AuSi	5.25	10.8	-
CaF_2_	AuC_2_	5.19	9.5	2.73
	AuN_2_	5.14	8.5	2.74
	AuO_2_	5.18	9.3	3.39
	AuSi_2_	5.90	24.5	-
