# Supplementary material for: Bottom-up formation of robust gold carbide
Source: Sci Rep. 2015 Mar 16;5:8891. doi: 10.1038/srep08891 (PMC5390915; doi:10.1038/srep08891)
Supplement: Supplementary Information [file srep08891-s6.pdf]

Bottom-up formation of robust gold carbide

Supplemental information

Benedikt Westenfelder[1], Johannes Biskupek[2*], Jannik C. Meyer[3], Simon Kurasch[2], Xiaohang Lin[4], Ferdinand Scholz[1], Axel Gross[4], and Ute Kaiser[2]

[1] Institute of Optoelectronics, Ulm University, 89081 Ulm, Germany

[2]Central Facility of Electron Microscopy, Ulm University, 89081 Ulm, Germany

[3]Department of Physics, University of Vienna, 1090 Vienna, Austria

[4]Institute of Theoretical Chemistry, Ulm University, 89081 Ulm, Germany

*corresponding author' s email: westbenedikt@gmail.com

In the following, we describe figures and image sequences to which we have been referred in the main text.

Growth on facets, on edges and crystallization:

The growth of AuC crystals has been observed for every possible sort of nucleation site, i.e. on the facets of gold particles, on the edges of defective or holey graphene and even on the boundaries of fullerene agglomerations (Figures S1 to S5). Defective two- and three-dimensional carbon structures formed by heat and electron irradiation provide active sources of atomic carbon under electron irradiation. Some sequences reveal, that the growing AuC structures do not only collect diffusing gold atoms but seemingly even `eat' fullerenes (sequence M1b with the corresponding snapshots in Figure S2 or regions of holey graphene (sequences M2 and M3 with the corresponding snapshots in Figures S4 and S5. The real time between each captured frame is 2 s. The frame rate of the final sequences is given in the figure captions.

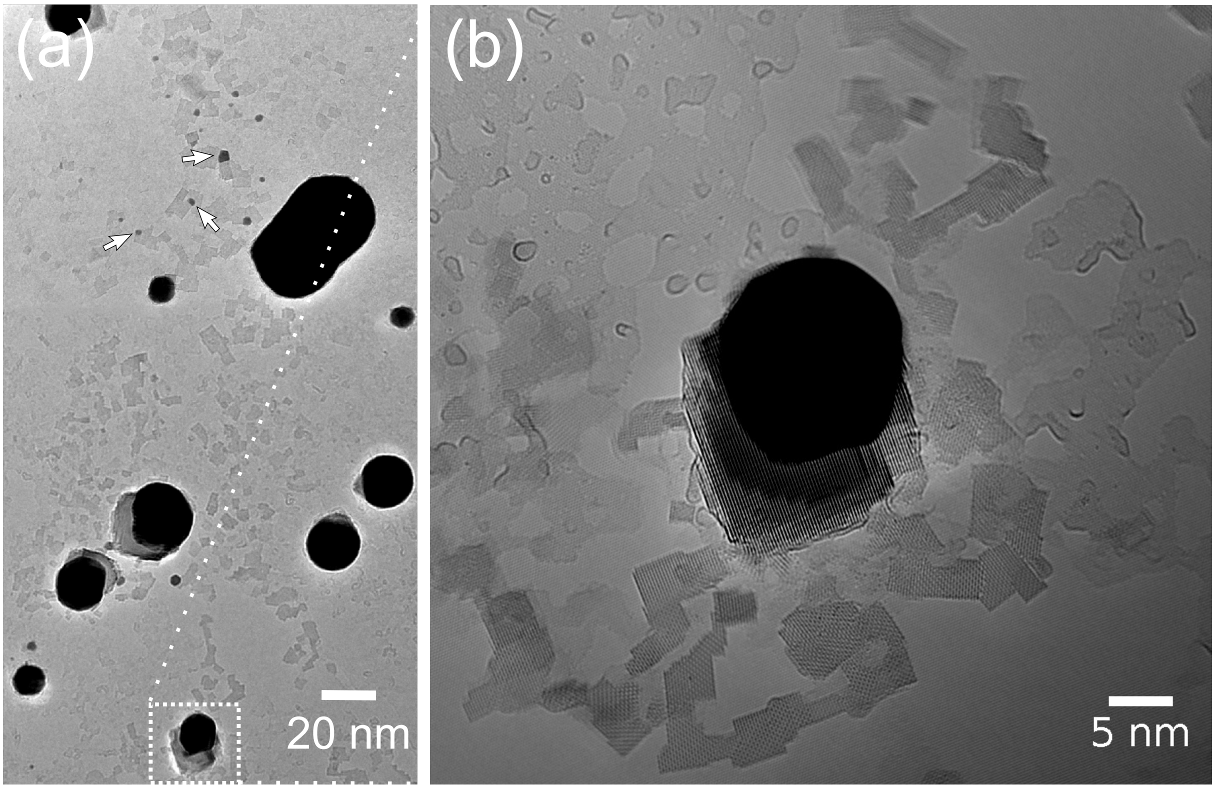

Figure S1. (a) Overview of sample location were the image sequence M1a has been captured. The exact location is indicated by a dotted quare. Tiny seed crystals for AuC structures are indicated by arrows. (b) Snapshot of the sequence M1a. The frame rate is 20 fps.

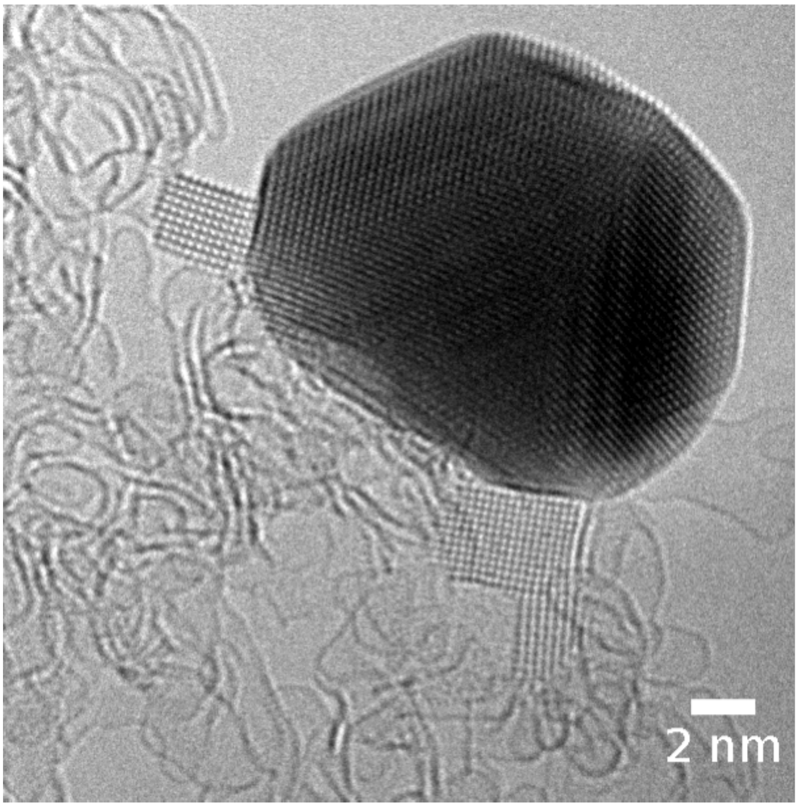

Figure S2. Snapshot of sequence M1b: A tiny AuC cube forms on the upper left facet. A second one is growing on the lower right facet. Both seemingly eat the fullerenes. The frame rate is 10 fps.

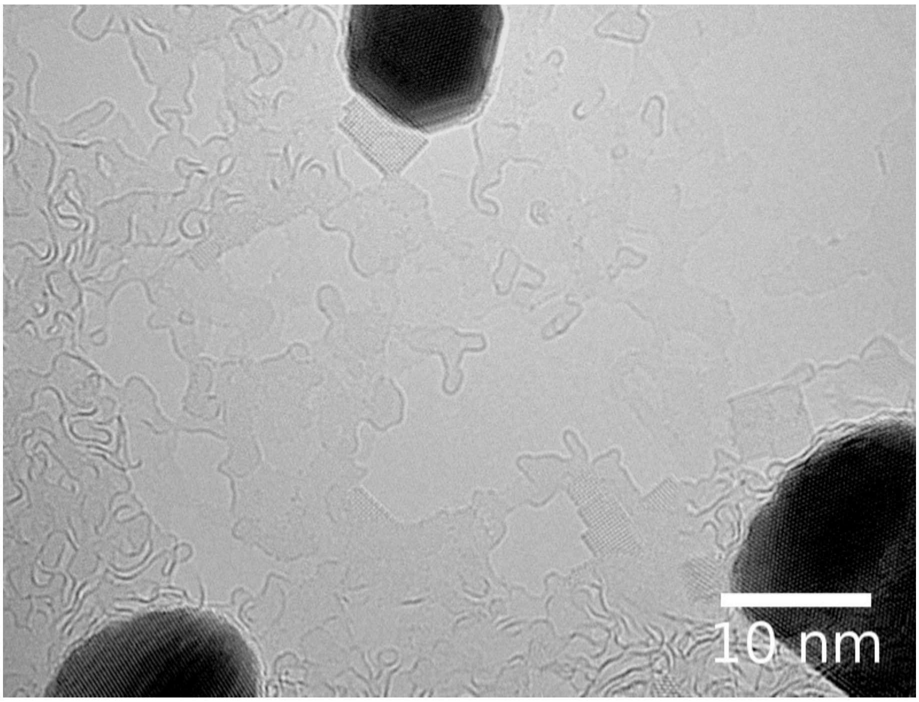

Figure S3. AuC cubes grown on Au particles and at the edges of partly crystallized layers of amorphous carbon.

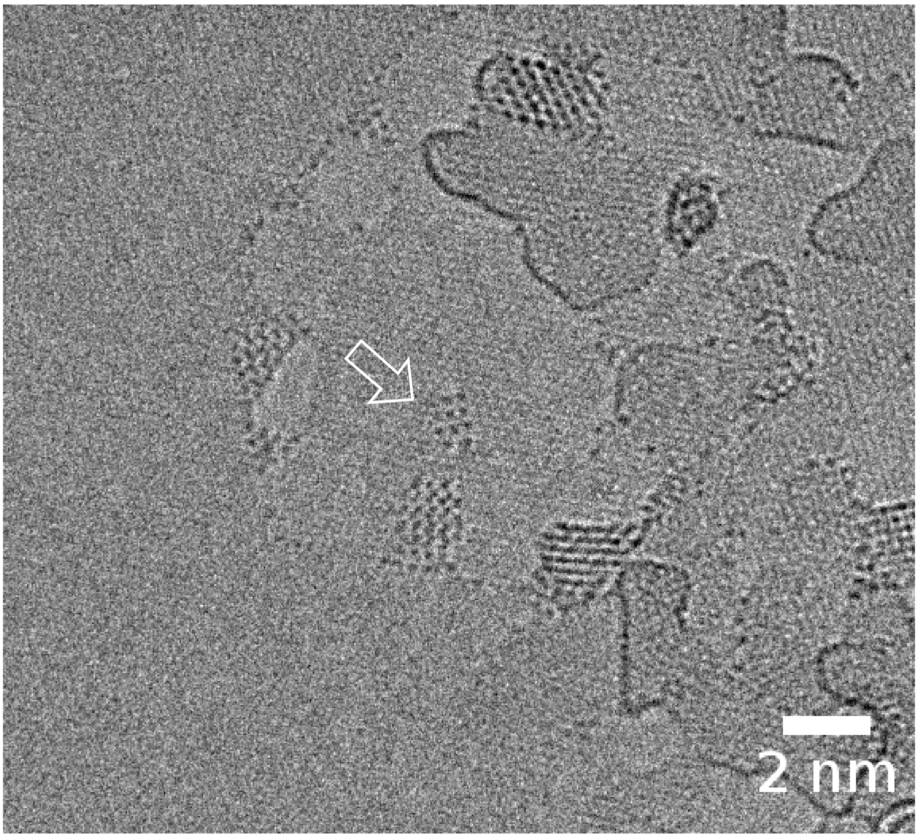

Figure S4. Snapshot of sequence M2: Growth and decomposition of tiny AuC crystals under increased electron dose of $3 \cdot 10^6$ *e-/(*nm^2s). Gold atoms apparently `eat' entire regions of holey graphene layers and form new AuC crystals. A tiny AuC matrix consisting of 8 to 10 visible atoms has formed as indicated by an arrow after 21 seconds. The frame rate is 10 fps.

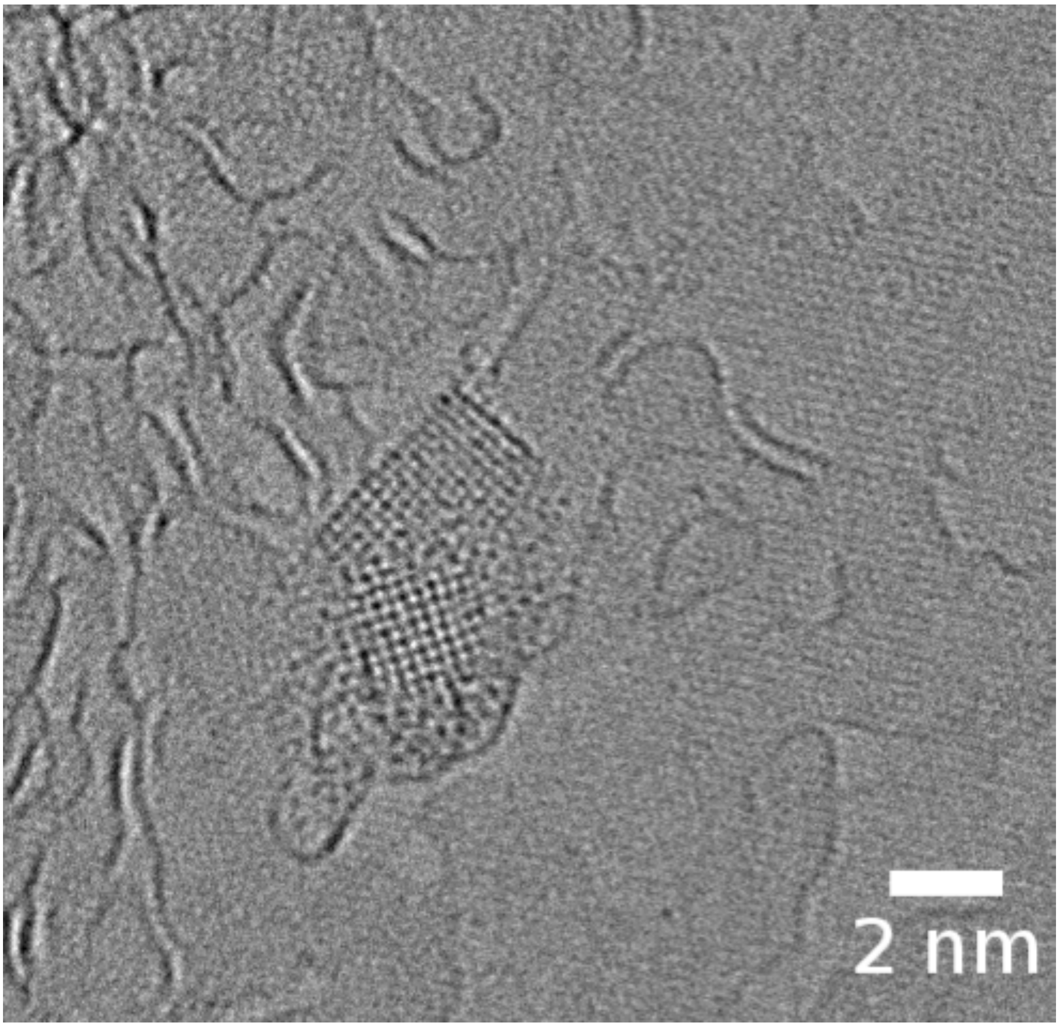

Figure S5. Snapshot of sequence M3: Crystallization of an amorphous huddle consisting of gold and carbon atoms. The huddle indicated by an arrow is present from the beginning of the sequence. With the beginning of the nucleation and growth of tiny AuC crystals at the edges of partly crystallized layers of amorphous carbon, two further arrows appear after 21 and 26 seconds indicating the nucleation of two new crystals. The frame rate is 10 fps.

Thermal stability of the AuC particles:

The AuC structures have been observed to exhibit a very large thermal stability as demonstrated by Figure S6 and the sequence M4 (snapshot in Figure S7).

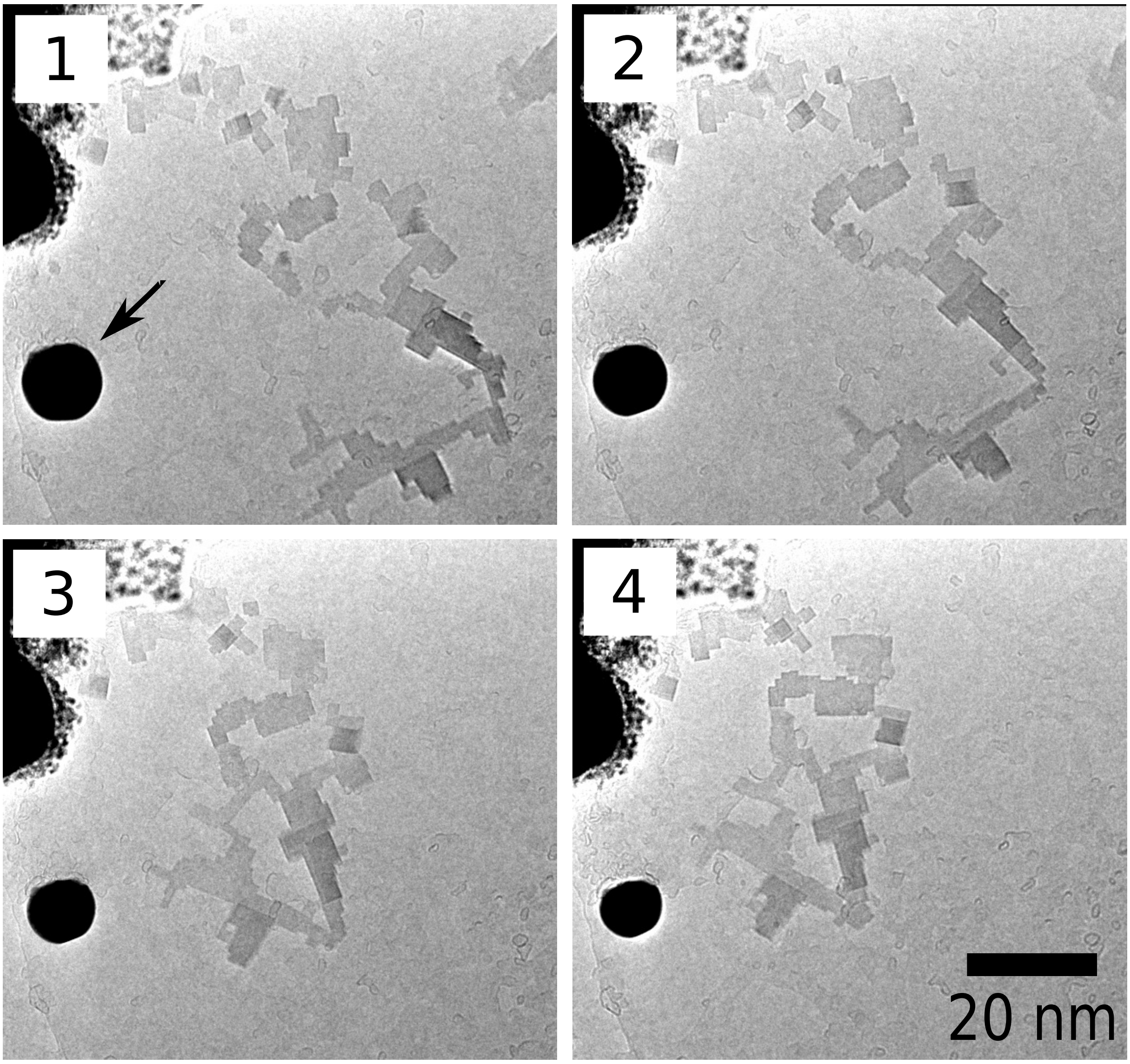

Figure S6. A gold particle larger than 10 nm (indicated by an arrow) starts to evaporate. This indicates a temperature of at least 1300 K. Interestingly, the somewhat smaller AuC cubes still move around but do not change their size and even preserve their crystalline structure. This image series was taken from a sequence lasting 60 seconds.

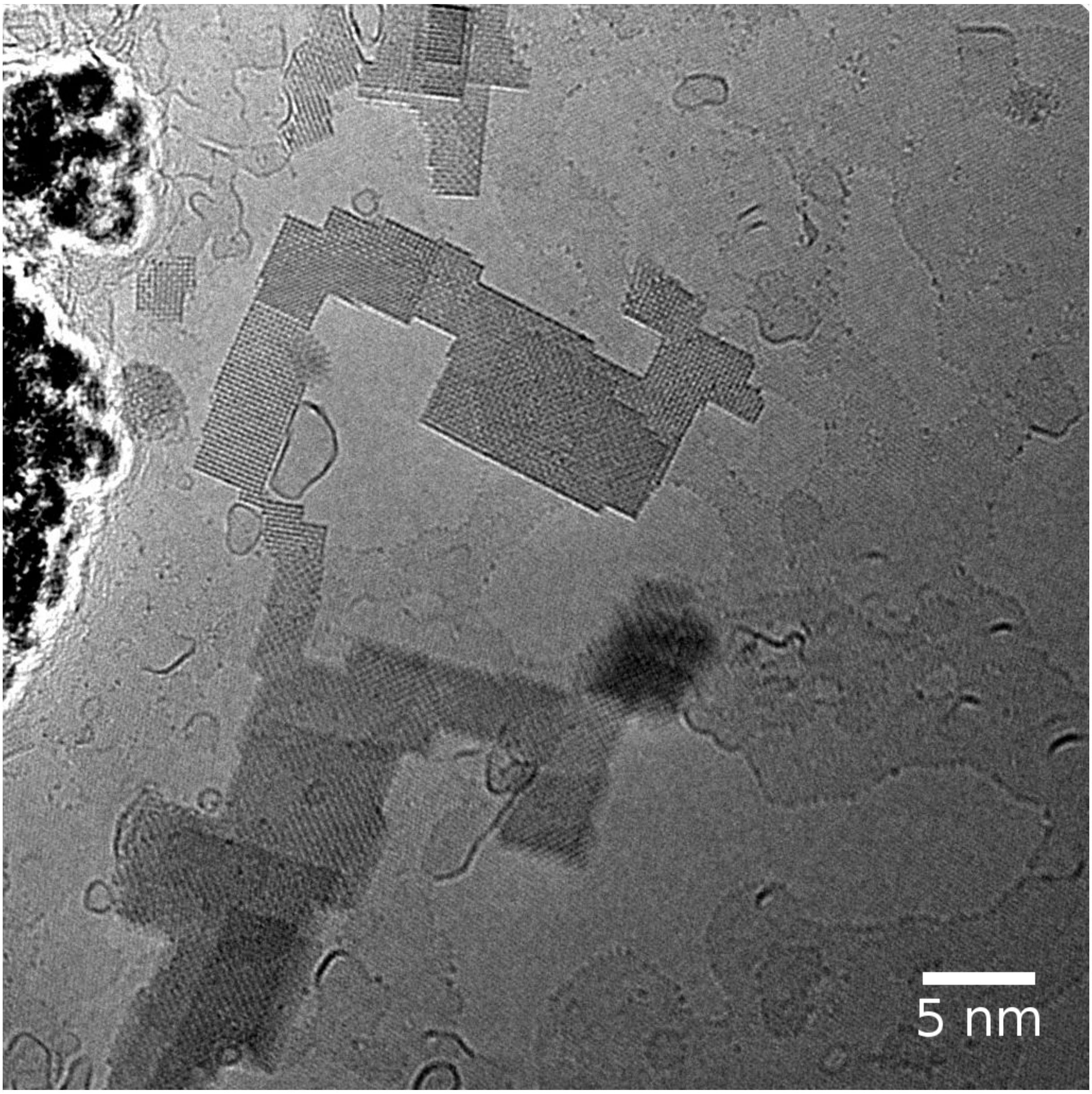

Figure S7. Snapshot of sequence M4: The temperature has further increased via increasing the electrical current through the multilayer graphene sheet. Finally, also the AuC starts to decompose. The frame rate is 10 fps.

Particles analyzed by EELS:

We performed local electron energy loss spectroscopy (EELS) to clarify the presence or absence of a possible second elements in our AuC structures. Figure S8 shows the AuC crystal that has been analyzed.

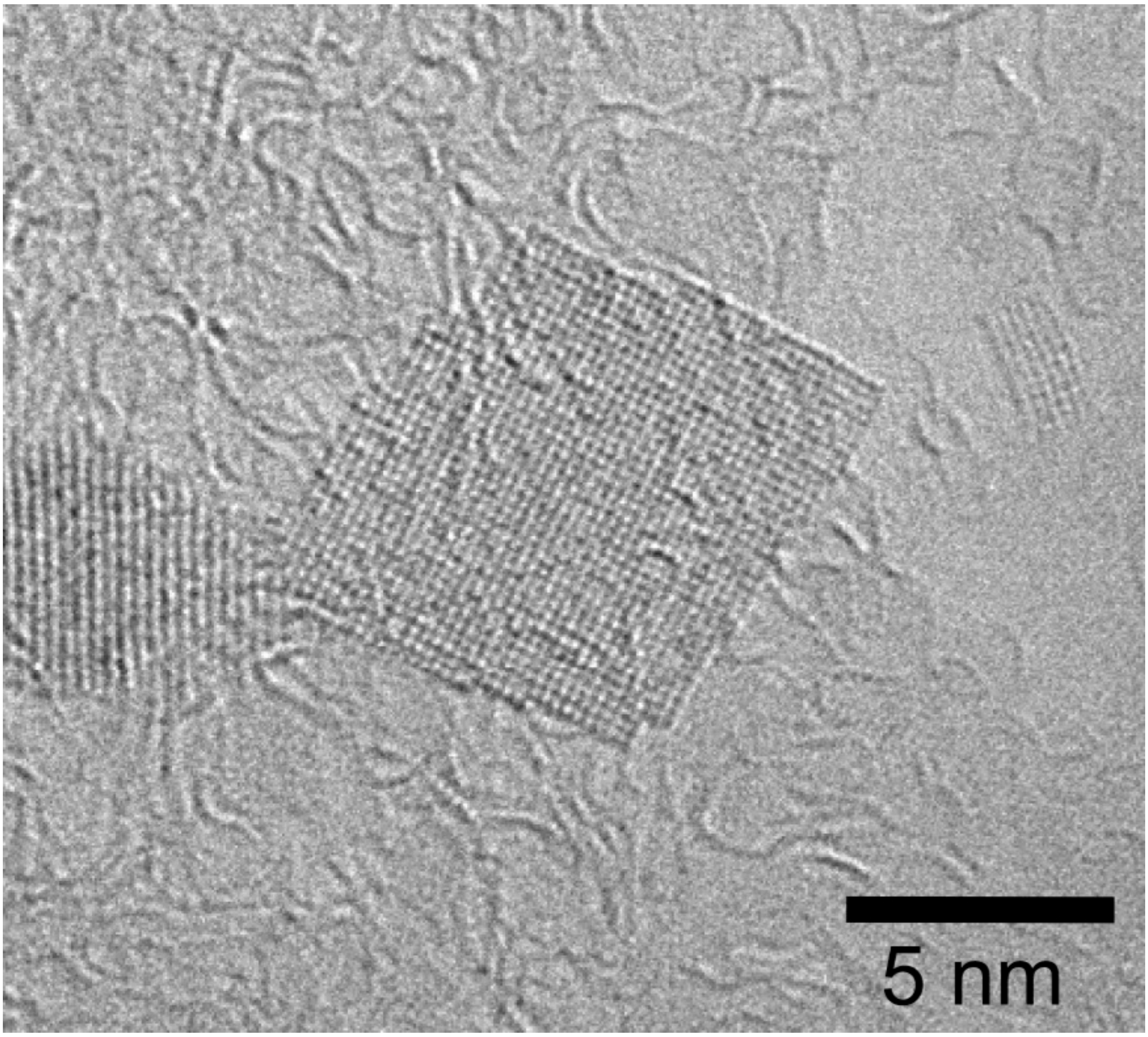

Figure S8. Particle that has been analyzed via EELS under a beam diameter of approximately 5nm.

Image simulations

We simulated TEM images for the AuC crystals in order to compare the most reasonable crystal structures (ZnS versus NaCl). Considering noise and the underlying graphene substrate, light elements like carbon embedded in the gold fcc lattice are practically invisible for both structures. Only in case of heavier elements like silicon, we would expect to reveal the lack of a fourfold symmetry in the simulated (100) projection of ZnS (Figure S9). The images are obtained via the multi-slice program MUSLI [1].)

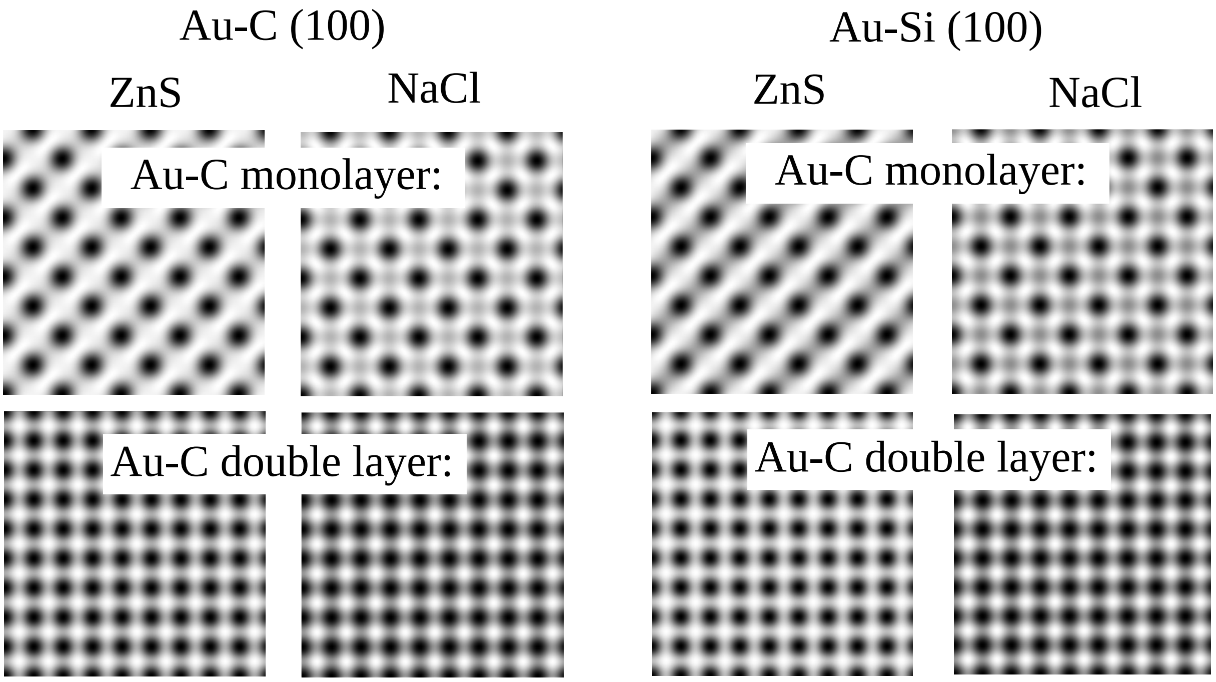

Figure S9. Simulated TEM images in order to compare the most reasonable crystal structures. Image simulation conditions: 80 kV, C_S=0.02 mm, df=-10 nm.

Additional data obtained by the DFT calculations:

The last discussion in the main article deals with the comparison of possibly formed gold compounds. In one regard we compared different surface terminations for an Au-C compound with sodium chloride (NaCl) and zinc blende (ZnS) structure. Here, we exemplify shortly the data obtained by our density functional theory (DFT) calculations: Figure S10 shows the formation energy of gold carbide in dependence of the number of atomic planes. Here, we have to differentiate between two situations:

- In the first case, the atomic planes which belong to a certain crystallographic orientation are occupied exclusively by gold *or* carbon atoms (gold and carbon atomic layers are alternately stacked). This is valid for ZnS(100), ZnS(111) and NaCl(111). In Figure S10 all odd numbers correspond to the layers which are occupied by gold atoms. If we consider this, we can conclude that a surface terminated with gold atoms is energetically preferred.
- In the second case, the atomic planes are always equally occupied by gold and carbon atoms. This is valid for NaCl(100), NaCl(110) and ZnS(110).

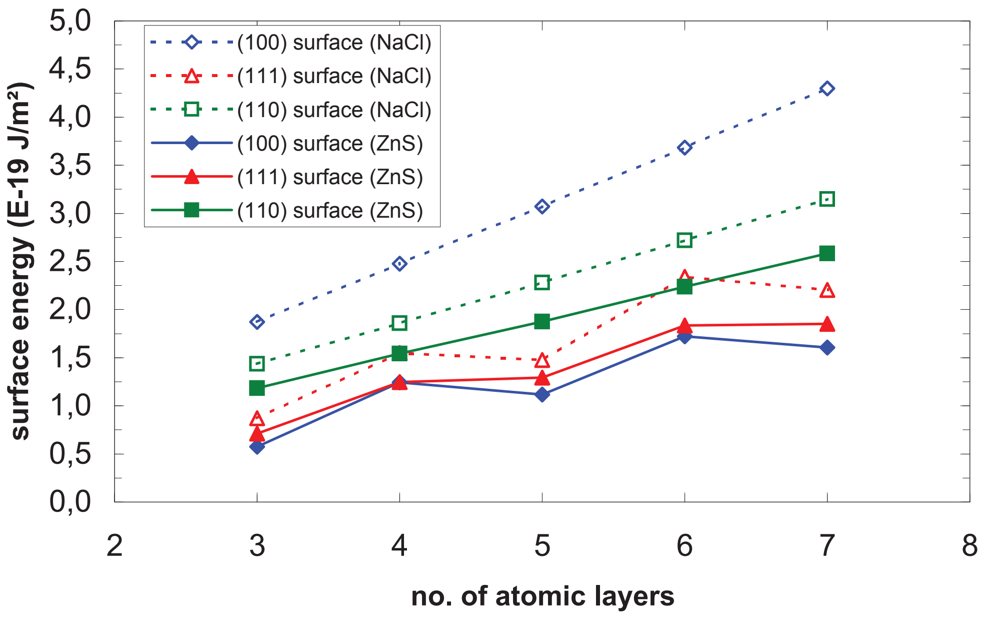

Figure S10. Formation energy in dependence of the number of atomic planes

It follows: if we consider an experimentally observed crystal consisting of three layers of gold atoms and with (100) surface termination, we have to compare the NaCl(100) formation energy for 3 atomic layers with the ZnS(100) formation energy for 5 atomic layers. Finally, we find from Figure S10

that the formation of the (100) plane is the most favorable one in case of ZnS but the most unfavorable one in case of NaCl.

The comparison between the density of states of the isolated cluster and the one of the interacting system (model presented in Figure S11a) shows that hardly any AuC-related peaks are shifted (Figure S11b). The total density of states can rather be regarded as a superposition of the densities of the AuC cluster and the graphene substrate. This indicates that there is a weak chemical interaction between AuC and graphene.

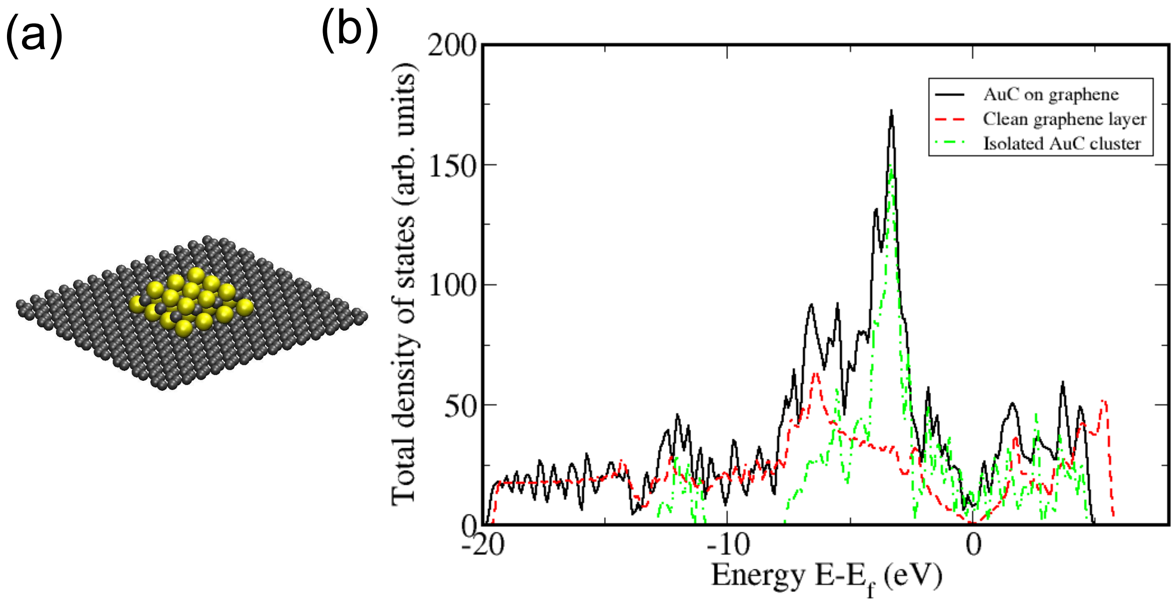

Figure S11. (a) Structure of the AuC cluster on graphene used to determine the interaction between AuC and graphene; b) Total density of states of the AuC cluster on graphene and of the pure graphene sheet and the isolated cluster.
